# Prevalence of herpes simplex virus types 1 and 2 antibodies among individuals screened in a tertiary hospital in the Eastern province of Saudi Arabia

**DOI:** 10.25122/jml-2022-0046

**Published:** 2022-10

**Authors:** Khaled Riyad Alkharsah, Nahid Hussein Wanni, Raghad Alsaffar, Reem Al Dossary, Obeid Eltreifi Obeid, Nourah Al Qahtani, Basavaraj Channabasappa Hunasemarada, Ayman Abdelmoamen El-Badry

**Affiliations:** 1 Department of Microbiology, College of Medicine, Imam Abdulrahman Bin Faisal University, Dammam, Saudi Arabia; 2 Department of Obstetrics and Gynecology, College of Medicine, Imam Abdulrahman Bin Faisal University, Dammam, Saudi Arabia

**Keywords:** herpes simplex virus, seroprevalence, HSV-1, HSV-2, Saudi Arabia

## Abstract

The epidemiology of herpes simplex virus (HSV) infections varies among populations depending on their demographic characteristics and exposure. This study describes the prevalence of HSV 1/2 IgG and IgM antibodies among individuals over a period of 5 years. A retrospective study was conducted to collect data on cases tested for HSV-1 and HSV-2 IgG and IgM antibodies for different medical conditions over five years between January 2014 and December 2018. 620 samples were tested for HSV 1/2 IgG and IgM during the study period. The total HSV seropositivity in the study population was 68% (422/620). The total seropositivity excluding children below 6 months of age was 65.3% (313/479). HSV-IgG seropositivity was significantly higher in married individuals (p<0.001, 95% CI 1.61–3.69). The HSV IgG seropositivity was significantly higher in children under the age of 6 months (N=109, 77.30%) than in children between 7 and 24 months old (27.6%) (Chi-square for linear trend, p<0.001), and it then tends to increase with age more than 24 months (Chi-square for linear trend, p=0.011). Eleven children showed laboratory evidence of recent HSV infection (6.2%) as indicated by HSV IgM antibodies and had diverse clinical conditions. HSV infection is highly prevalent in the Eastern Province of Saudi Arabia. Infection is most probably acquired during early childhood, and the tendency increases with age. However, a significant number of mothers are at risk of infection and transferring the infection to their fetuses.

## INTRODUCTION

Herpes Simplex virus (HSV) is a large enveloped DNA virus of icosahedral symmetry which belongs to the family *Herpesviridae*. Primary infection is usually either asymptomatic or presented as skin or mucosal lesions [1, 2]. After that, the virus persists latently for life in the ganglia near the site of the primary infection.

There are two types of HSV. HSV-1 usually causes oral sores, often referred to as fever blisters or cold sores and establishes persistent infection in the trigeminal ganglia, while HSV-2 typically causes sores in the genital region or rectum and establishes persistent infection in the sacral ganglia. Transmission of HSV-1 primarily occurs during childhood or adolescence through nonsexual contact. HSV-2 transmission usually occurs through sexual contact in adults, while infants get infected through their infected mothers [3]. Most neonatal HSV infections are acquired during delivery, while the minority are caused by postnatal viral acquisition [4].

HSV infections are widespread among humans worldwide, and because herpes is a lifelong infection, its estimated prevalence increases with age [5, 6]. It is estimated that 67% of people below 50 years old have HSV-1 infection worldwide, and 13% of people between 15–49 years have HSV-2 infection [7]. The prevalence of HSV-1 and HSV-2 infection in the United States was reported as 47.8% and 11.9%, respectively [8]. While across Europe there is an appreciable difference in the seroepidemiology of HSV-1 and HSV-2, women appeared to have higher HSV-2 seropositivity than men [9]. In Africa, the incidence of HSV-2 is 20–80% in females, and 10–50% in males, while HSV-1 incidence is equivalent in women and men by about 50% [6]. In the Middle East and North Africa, the seroprevalence of HSV-1 showed that 65% of children and 90% of adults had been exposed to this infection, most often during childhood [10]. In Saudi Arabia, one of the earliest studies done in 1986 showed that 92% of pregnant women are HSV-1 IgG positive and 6.3% are HSV-2 IgG positive [11]. This was followed by another study on 770 individuals showing HSV-1 and HSV-2 seroprevalence of 89.5% and 3%, respectively [12]. A later study in 2015 also showed that HSV-1 infection is widespread in Saudi Arabia and most probably acquired before adulthood, while HSV-2 prevalence is very low and acquired in adulthood and increased with age [13].

Many people with HSV-1 and HSV-2 infections do not know they are infected because most infections are subclinical [14]. Therefore, recognizing the viral seroepidemiology within populations and employing appropriate public health procedures would be significant to control HSV infection. Additionally, the disease burden of this infection has drawn the attention of the World Health Organization (WHO) and global partners to focus on understanding the epidemiology of the virus and developing an HSV vaccine [15].

HSV infection results in the production of lifelong antibodies. Immunological laboratory tests detect previous asymptomatic HSV-1 or HSV-2 infections or identify current infections in symptomatic patients [16, 17]. HSV-1 and HSV-2 have high genetic similarities, which leads to an antigen resemblance between both serotypes [18]. As a result, antibodies produced due to infection with one serotype extensively cross-react with the other serotype.

Determining the specific IgG antibodies against HSV-2 and HSV-1 is a reliable estimation of their population-based seroprevalence [19]. The prevalence of HSV antibodies has been reported in countries across the globe and has been found to vary by place and population [20]. The aim of this study was to provide an update on HSV antibody prevalence among the population of the Eastern Province in Saudi Arabia and to characterize the infection's age distribution, gender, marital status and nationality over 5 years.

## Material and Methods

### Study settings

A retrospective study was conducted at King Fahd Hospital of the University in Al Khobar city, Eastern Province of Saudi Arabia. This tertiary hospital accommodates more than 600 beds and serves more than 5 million populations in the Eastern Province.

The study comprises cases tested for HSV antibodies for different medical conditions and checkups over five years between January 2014 and December 2018. Data were obtained from the archived medical records at the hospital. A total of 620 samples (over 5 years) were tested for HSV 1/2 IgG and IgM antibodies. The total number of samples excluding patients less than 6 months of age was 479, and the total number of samples excluding patients less than 2 years of age was 178.

### HSV antibody testing

HSV-1 and HSV-2 IgG and IgM antibodies were previously measured at the immunology laboratory of the hospital using LIAISON HSV 1/2 IgG and LIAISON HSV 1/2 IgM kits, respectively (Diasorin, Via Crescentino, Saluggia, Italy). Both kits employ the chemiluminescence immunoassay (CLIA) technique and are used on the Liason machine (Diasorin, Via Crescentino, Saluggia, Italy). A result of less than 9 standard units was considered negative, while a result of more than 11 standard units was considered positive. A result of 9–11 standard units was considered a gray zone and would require to be repeated on another sample. According to the kit's booklet, the sensitivity and specificity of the assays are more than 95%.

The presence of IgG alone indicates previous exposure to the virus or maternal antibodies in infants. The presence of IgM alone indicates a current exposure to the virus, while the presence of both antibodies indicates recent exposure to the virus.

### Statistical analysis

The data were tabulated in Excel spreadsheets, calculating the frequencies. The statistical associations were investigated using the OpenEPI website (Emory University, Atlanta, Georgia, United States). The Chi-square test was used to calculate the association of HSV antibody detection with gender, marital status, nationality, and child status. At the same time, the chi-square for linear trend was used to calculate the association with age groups. Post-hoc analysis was used to calculate the association with disease groups. A p-value of less than 0.05 was considered significant. Because of the wide variety of disease conditions among the study population, the disease conditions were grouped into system disorders or condition disorders.

## Results

**Figure 1:**
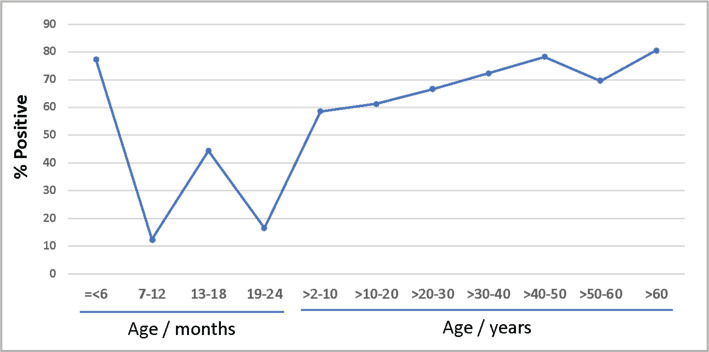
Percentage of individuals in the study population positive for HSV IgG antibodies with age.

The total HSV seropositivity in the study population was 68% (422/620). The total seropositivity excluding children below 6 months of age was 65.3% (313/479). The demographic data for all individuals tested for HSV IgG antibodies is illustrated in Table 1. HSV-IgG seropositivity was significantly higher in married patients (p<0.001, 95% CI 1.61–3.69) and tends to increase with age (Chi-square for linear trend, p=0.011) (Table 1 and Figure 1). There was no significant difference in HSV IgG levels between males and females (p=0.408, 95% CI 0.61–1.22) and between Saudis and expatriates (p=0.426, 95% CI 0.55–1.30) (Table 1).

**Table 1:** Demographic data of all patients tested for HSV-IgG over 5 years.

	Negative		Positive		Total	P-value
	N=198	%	N=422	%		
Gender*						
Females	103	30.56	234	69.44	337	0.408
Male	95	33.69	187	66.31	282	
Marital status**						
Single	158	37.09	268	62.91	426	0.000
Married	36	19.57	148	80.43	184	
**Nationality**						
Saudi	159	31.24	350	68.76	509	0.426
Non-Saudi	39	35.14	72	64.86	111	
**Age groups (year) $**						
0–10	80	35.71	144	64.29	224	0.011
>10–20	22	38.60	35	61.40	57	
>20–30	41	33.33	82	66.67	123	
>30–40	26	27.66	68	72.34	94	
>40–50	10	21.74	36	78.26	46	
>50–60	10	30.30	23	69.70	33	
>60	7	19.44	29	80.56	36	

* – 1 sample is missing; ** – 10 samples are missing; $ – 7 samples are missing. P-value for Chi-square for linear trend.

When the demographic data and the disease conditions were analyzed for children under 2 years tested for HSV IgG antibodies (N=178), we found that the overall seropositivity of HSV among all children was 65.7% (117/178) (Table 2). The HSV IgG seropositivity was significantly higher in children under 6 months (N=109, 77.30%), and the level of HSV antibodies declined after that (Chi-square for linear trend, p<0.001) (Table 2 and Figure 1). The prevalence of HSV IgG antibodies was 27.6% (8/29) among children between 7 and 24 months old. There was no significant association between certain diseases and HSV IgG antibodies. However, children with skin disorders were less likely to have HSV IgG antibodies (p=0.003) (Table 2).

**Table 2:** Demographic data of patients under 2 years of age tested for HSV IgG antibody.

	Negative		Positive		Total	P-value
	N=61	%	N=117	%	N=178	
Gender*						
Females	26	30.23	60	69.77	86	0.255
Male	35	38.46	56	61.54	91	
**Age groups (months)$**						
≤6	32	22.70	109	77.30	141	<0.000
7–12	14	87.50	2	12.50	16	
13–18	5	55.56	4	44.44	9	
19–24	10	83.33	2	16.67	12	
**Diseases**						
Autoimmune disorders	0	0.00	1	100.00	1	1
Bleeding disorder	1	16.67	5	83.33	6	0.658
Neurological disorders	2	12.50	14	87.50	16	0.087
Growth and developmental disorders	14	26.42	39	73.58	53	0.204
Congenital disorders	1	11.11	8	88.89	9	0.144
Infectious disorders	26	43.33	34	56.67	60	0.074
Gastrointestinal and hepatobiliary disease	6	28.57	14	66.67	21	0.691
Skin disorders	8	80.00	2	20.00	10	0.003
Genetic disorder	2	100.00	0	0.00	2	0.232
**Child Status**						
Discharged	57	35.85	102	64.15	159	0.200
Died	4	21.05	15	78.95	19	

* – 1 sample is missing.

As shown in Table 3, most HSV-IgG-positive children under 2 years were suffering from growth and developmental disorders, infectious diseases, neurological disorders, and gastrointestinal and hepatobiliary disorders. Other clinical conditions included congenital diseases, bleeding disorders and others (Table 3).

**Table 3:** All IgG-positive cases under 2 years classified according to age in months and disease groups as a reason for testing.

Disease grouping	≤6	7–12	13–18	19–24	Total
Autoimmune diseases	0	0	0	1	1
Bleeding disorder	5	0	0	0	5
Neurological disorders	12	1	1	0	14
Growth & developmental disorders	39	0	0	0	39
Congenital disorders	8	0	0	0	8
Infectious disorders	30	1	3	0	34
Gastrointestinal and Hepatobiliary disease	14	0	0	0	14
Skin disorders	1	0	0	1	2
Genetic disorder	2	0	0	0	2
Total	**109**	**2**	**4**	**2**	**117**

Of the 178 children under the age of 2 years, 11 showed laboratory evidence of recent HSV infection (6.2%) as indicated by HSV IgM antibodies (Table 4). Eight of the HSV-IgM-positive children were also positive for HSV IgG antibodies (72.7%) (Table 4). The description of age, gender, nationality, and clinical disease presentations of the 11 IgM-positive patients is detailed in Table 4.

**Table 4:** Description of cases under 2 years old and positive for HSV-IgM.

IgG	Sex	Nationality	Age months	Disease group	Disease condition
Neg.	F	Saudi	22	Dermatological condition	Post-inflammatory hypopigmentation after herpes simplex
Pos.	M	Saudi	1.5	Fever	Neonatal fever to r/o sepsis
Neg.	M	American	21	Fever	Prolonged fever for investigation
Pos.	M	Saudi	2	Hyperbilirubinemia	Premature, umbilical hernia and direct hyperbilirubinemia
Neg.	M	Saudi	21	Infection	Herpetic eye disease
Pos.	F	Saudi	15	Infection	Stomatitis
Pos.	F	Saudi	At birth	IUGR	Premature with IUGR (mother-positive IgG)
Pos.	F	Saudi	At birth	IUGR	Premature with IUGR
Pos.	F	Saudi	3	Jaundice	Cholestatic jaundice
Pos.	F	Saudi	5.5	Pneumonia	Bronchopneumonia
Pos.	F	Saudi	19	Dermatological condition	Dermatological condition not specified

IUGR – Intrauterine growth restriction.

## Discussion

This study describes the prevalence of HSV 1/2 IgG and IgM antibodies among 620 individuals over 5 years. The main finding of our study showed a seroprevalence of HSV antibodies in 68% of the tested population, while it was 65.3% among individuals above 6 months old. These results indicate a lower HSV antibody seroprevalence than previously reported by other studies from Saudi Arabia [13, 21–23]. However, it could be attributed to the involvement of children in the analysis in our study, unlike other studies, which focused on healthy adults or pregnant women. In the Middle East and North Africa (MENA) region, the seroprevalence was 65.2% in children and 91.5% in adults [10].

We did not find a significant difference in HSV IgG seroprevalence between males and females or between Saudis and expatriates. On the other hand, the HSV-IgG seropositivity was significantly higher in married individuals, which agrees with Memish et al. and others [13, 24, 25].

As shown in Figure 1, there was a clear tendency of increased HSV antibodies seroprevalence with age. The highest prevalence during childhood was among children under 6 months of age (77.3%). These antibodies are most likely the maternal antibodies transmitted to the fetus during pregnancy. There was a decline in the level of HSV antibodies, indicating the gradual loss of maternal antibodies to about 16% by the age of 2 years. It is not possible to differentiate between HSV maternal antibodies and antibodies due to infection between 7 months and 2 years. However, a large proportion of children under 2 years are at risk of acquiring HSV infection. The level of HSV antibodies increased gradually with age to reach a maximum in the highest age group reflecting a cumulative exposure risk. Our results are in line with previous estimates that 90% of all individuals possess antibodies to HSV type 1 by adolescence and adulthood age (20–30 & 30–40 years), reflecting the cumulative risk of exposure [20, 26].

Of the 178 children under 2 years, eleven (6.2%) showed the presence of serum IgM, and 8 (72.7%) showed simultaneous presence of serum IgG. The presence of serum IgM in children and neonates is an important indicator for diagnosing recent and intrauterine infections. A recent infection during childhood is more likely associated with poor hygienic and socioeconomic conditions [21, 27]. Active HSV lesions or prodromal symptoms at the onset of delivery increase the risk of viral exposure to the neonate [28]. In order to avoid neonatal herpes cases, identification of the mother at risk is essential. The estimated rates of neonatal herpes simplex virus infection vary across different regions of the world [20]. Worldwide, an estimated 75% of neonatal HSV cases are caused by HSV-2 and 25% by HSV-1 [26].

In our study cohort, 117 children under 2 years were positive for HSV IgG antibodies. No specific clinical presentation was associated with HSV seropositivity. However, those with positive serum IgM had infections, fever, dermatological conditions, and jaundice. Two infants had positive serum IgM directly at birth, indicating an intrauterine infection and was associated with intrauterine growth restriction.

HSV infections in neonates and infants have a variable presentation and may simulate other neonatal infectious conditions. As a result, they may go unrecognized or be attributed to another disease process. Disseminated HSV infections are often fatal, and survivors could have substantial neurological sequelae.

In this study, there was no significant difference in the HSV antibody seroprevalence between male and female children below two years of age. However, seven out of eleven (63.6%) children with HSV IgM antibodies were females. It can be attributed to the very small sample size. Nevertheless, similar findings were reported in Europe and the USA where female children were more likely to be HSV seropositive than males [9, 29, 30]. A recent study including 190 pregnant women found anti-HSV-2 IgG antibodies in only 0.5% of the study population [23], which may reflect a very low prevalence of HSV-2 among the Saudi population.

## Conclusions

Our study presented the serological prevalence of HSV IgG antibodies among different age groups from the Eastern Province of Saudi Arabia. However, we could not differentiate between HSV-1 and HSV-2 infection.

## Data Availability

Further data is available from the corresponding author on reasonable request.

## Personal thanks

The authors would like to thank the IT department at King Fahd Hospital of the University (KFHU) for helping with data retrieval. We also would like to thank the staff at the Immunology Laboratory at the KFHU for their help in obtaining some data from their records.
